# Efficient calculation of SAMPL4 hydration free energies using OMEGA, SZYBKI, QUACPAC, and Zap TK

**DOI:** 10.1007/s10822-014-9720-8

**Published:** 2014-03-16

**Authors:** Benjamin A. Ellingson, Matthew T. Geballe, Stanislaw Wlodek, Christopher I. Bayly, A. Geoffrey Skillman, Anthony Nicholls

**Affiliations:** OpenEye Scientific Sofware, 9 Bisbee Court, Suite D, Santa Fe, NM 87508 USA

**Keywords:** SAMPL, SAMPL4, OpenEye, OMEGA, QUACPAC, SZYBKI, Zap, Freeform, Poisson–Boltzmann, PB, PBSA, AM1BCC, Solvation, Hydration free energy

## Abstract

Several submissions for the SAMPL4 hydration free energy set were calculated using OpenEye tools, including many that were among the top performing submissions. All of our best submissions used AM1BCC charges and Poisson–Boltzmann solvation. Three submissions used a single conformer for calculating the hydration free energy and all performed very well with mean unsigned errors ranging from 0.94 to 1.08 kcal/mol. These calculations were very fast, only requiring 0.5–2.0 s per molecule. We observed that our two single-conformer methodologies have different types of failure cases and that these differences could be exploited for determining when the methods are likely to have substantial errors.

**Electronic supplementary material:**

The online version of this article (doi:10.1007/s10822-014-9720-8) contains supplementary material, which is available to authorized users.

## Introduction

In this paper, we discuss our submissions to the hydration free energy portion of the SAMPL4 challenge [1, 2]. The SAMPL challenge is an informal prospective challenge where participants do not receive the experimental results until after their modeling results have been submitted. Although the effects of solvation are extremely important to organic and medicinal chemistry, new experimental results are rarely published. Studies of solvation models are almost entirely retrospective due to the lack of new experimental data, thus omitting the prediction step of the scientific method and making models susceptible to over-fitting with parameters. It is unreasonable to expect accurate results when modeling complicated interactions with solvation effects, such as protein–ligand binding, when the scientific method has not been rigorously followed for the underlying models. The SAMPL challenge is informally prospective because the experimental data is only obscure, derived from publications without any indication in the title that hydration free energy data is available [3], whereas a truly prospective challenge would require newly measured data after predictions have been made. Peter Guthrie has been remarkably successful at gathering unseen data for the participating groups [2–6], thus making the SAMPL challenges effectively prospective.

Past submissions from the authors and collaborators [3, 5–11] have shown that the Zap TK Poisson–Boltzmann (PB) solver is competitive with the very best methods available for the prediction of hydration free energies. In the first SAMPL challenge, commonly referred to as SAMPL0 [3], the Poisson–Boltzmann surface area (PBSA) method with AM1BCC [12, 13] charges and ZAP9 radii [7, 8] yielded a root-mean-square (RMS) error of 1.87 kcal/mol. The SAMPL1 [8] hydration free energy set was exceptionally challenging due to large, highly polarizable molecules with multiple functional groups. PBSA with AM1BCC charges and ZAP9 radii yielded an RMS error of 2.44 kcal/mol, which was disappointingly large, yet was the lowest RMS error of any submission for that challenging dataset. The SAMPL2 challenge [7, 11] revealed the importance of conformer choice when using only a single conformer, with RMS errors for the same method as before ranging from 2.38 to 3.45 kcal/mol. The RMS error for this method in SAMPL3 was 2.72 kcal/mol [6], although a better choice of conformer may have improved results. Unfortunately, the methodology for finding the best gas-phase conformer, which was introduced in SAMPL1 and used in SAMPL4, was not used in SAMPL3. Additionally, the SAMPL3 dataset was specifically designed to investigate analogs with increasing numbers of chlorine atoms and should not be considered a general-purpose test of solvation methods.

In previous SAMPL challenges, we have investigated numerous variations and deviations from our standard calculation of PBSA with AM1BCC charges, such as partial charges derived from expensive density function theory calculations [8, 11], parameterization of radii for PBSA [7, 8], combinations of Cramer–Truhlar solvation and charges [14] with our methods [11], and investigations of conformer dependence and selection [7, 8, 11]. These past investigations have significantly impacted our best practices for solvation energy calculations, perhaps most importantly the parameterization of the ZAP9 radii [7, 8], which were used for all of our SAMPL4 submissions involving PBSA.

For the SAMPL4 challenge, we submitted variations of our mainstay calculation of PBSA with AM1BCC charges. Two of these have now been formalized in a new tool, *freeform*, which is distributed as part of the SZYBKI 1.8.0 package [15].

## Software

### Zap TK

All of our submissions are based on continuum solvation, often referred to as implicit solvation. With continuum solvation, the solvent is modeled as a dielectric continuum, whereas explicit solvation models use individual solvent molecules. Continuum solvation methods forgo individual solute–solvent interactions in exchange for effectively time-integrating over all of the solvent phase space. Our tool for calculating solvation energies is the Zap TK PB solver [16, 17]. When salt is not present or can reasonably be neglected, which is the case for the SAMPL challenge, the PB equation reduces to Poisson’s equation:

$$\nabla \epsilon \,({\varvec{r}})\,\nabla {\varphi }\,({\varvec{r}}) = -\rho\,({\varvec{r}})$$

where $\epsilon$ is the dielectric constant, *φ* is the potential, and *ρ* is the charge density, all of which are functions of the position vector, ***r***. Calculating solvation energies with this method requires very few parameters, and could be done with no parameters at all. Dielectric constants can be experimentally measured, atomic radii for creating the envelope of low dielectric inside the molecule can be determined from crystal structures, and the charge density can be determined from ab initio electronic structure calculations. However, optimizing the atomic radii of a few atom types significantly improves the results, as well as using a parameter to scale the surface area (SA) in order to include a hydrophobic term [3, 8]. The resulting ZAP9 parameters include 8 adjusted atomic radii and a SA factor of 6.3 calories per square angstrom.

The partial differential equation is solved on a grid, where the molecule is centered in the middle with several angstroms of grid points surrounding the surface of the molecule. For the aqueous phase, water is modeled by surrounding the molecule with a dielectric of 80. The internal dielectric is set to unity based on prior work [3], necessary given the atomic partial charging method we use. The transition from the internal to external dielectric is modeled using a smooth function based on the work of Grant et al. [17]. The charge density is approximated with partial charges at atom centers which have been calculated with QUACPAC [18] (described below).

Zap TK solves for the electrostatic potential, *φ*, which can then be used to calculate the electrostatic potential energy, *U*_*elec*_, from atom-centered partial charges, *ρ*, using the following equation.

$$U_{elec} = \frac{1}{2}\sum\limits_{partial \, charges} {\varphi \,(\varvec{r})\,\rho (\varvec{r})\,}$$

The hydration free energy can be modeled as the difference in energy between an external dielectric of 80, representing aqueous phase, and a uniform dielectric of one, representing gas phase, and then adding a hydrophobic term for non-electrostatic contributions. This hydrophobic term is calculated as a function of the SA, thus the commonly used acronym PBSA for the Poisson–Boltzmann surface area method.

### QUACPAC

The software package QUACPAC [18] from OpenEye was originally named from the phrase Quality Atomic Charges, Proton Assignment, and Canonicalization. The hydration free energies are defined for the neutral state of the given molecules, so the proton assignment functionality was not necessary for this particular challenge. However, in other systems where the movement of hydrogen is not restricted, the examination of tautomers and pKa states can be vitally important. OpenEye’s implementation of AM1BCC charges [12, 13] is distributed in the QUACPAC package, as well as various other partial charging methods. QUACPAC was used to generate partial charges for all of our SAMPL4 submissions.

### OMEGA

Conformers were generated using the OMEGA [19, 20] package from OpenEye. OMEGA is a model builder and torsion driver, yielding an energy-sorted ensemble of unique conformers. The default force field is MMFF94S [21] with intramolecular electrostatics removed, which tends to yield extended conformations due to the lack of strong attractive forces. Optionally, the standard MMFF94 [22–26] force field may be used, as was done with the Freeform submissions. OMEGA is highly customizable as is seen in the method descriptions below.

An initial implementation of hydrogen sampling was recently introduced in version 2.5.1 of the OMEGA package [20]. Previous versions of OMEGA only sampled heavy-atom locations and hydrogen atoms were set once prior to torsion driving using a heuristic. It has been noted in previous SAMPL challenges [7, 8] that the position of hydrogen atoms can significantly affect solvation. The new option samples multiple hydrogen positions for –OH, –SH, and amines while enumerating conformers and then sorts by force field energy. This allows the force field to determine which hydrogen position has the lowest energy for each conformation.

### SZYBKI

SZYBKI [15] is OpenEye’s implementation of the MMFF94 [22–26] and MMFF94S [21] force fields, and also includes other functionalities such as solvation in order to optimize structures and calculate properties. A new tool, *freeform*, marks a significant increase in the functionality of SZYBKI by including conformer generation and deduplication settings that have been customized for each specific mode of the application.

The *freeform* application currently has two modes, a solvation mode for the hydration free energy of a small molecule and a conformer mode for the free energy required to select one particular conformer out of the whole conformational ensemble in solution. The methods labeled as FreeformSolv below refer to running this application in the solvation mode, which is an implementation of the workflow for conformer selection and PBSA calculation originally introduced by Nicholls et al. [7, 8] in the SAMPL1 and SAMPL2 challenges.

## Methods

### FreeformSolv: submissions #565 and #566

The goal of this method is to generate a single conformer with the lowest-energy gas-phase geometry and then use PBSA to calculate the hydration free energy. The primary hypothesis of this submission is that the additional internal energy required for adopting the conformation with the lowest solvation energy is nearly equivalent to the increase in solvation energy associated with that conformation; therefore, calculating transfer energies with a single low-energy gas-phase conformation is an effective strategy. When this effect was directly studied in SAMPL1 using MMFF94 and PBSA, the difference between the change in total internal energy and solvation energy was within 1 kcal/mol for all 56 compounds [8].

These submissions used a pre-release beta version of the *freeform* application in solvation mode. The internal workflow is as follows:

1. The molecule is passed into the OMEGA algorithm with the following parameters:
  - Maximum number of output conformers is set to 200.
  - RMS threshold for duplicate removal is set to 0.6 angstroms.
  - Force field for the energy sorting of conformers is set to standard MMFF94.
  - The highest allowable energy for a conformer, i.e. the energy window, is set to 25 kcal/mol above the lowest energy conformer in the set.
2. The conformer set is then passed into a SZYBKI gas-phase optimization with default MMFF94 force field settings. The lowest energy conformer from this set is chosen for the solvation energy calculation.
3. AM1BCC charges are set on the single conformer. For submission #565, unsymmetrized charges were used. This variant of AM1BCC charges is equivalent to OECharges::AM1BCCNoSymSPt in the Quacpac toolkit, which assigns charges without optimization or symmetrizing topologically-equivalent atoms. For submission #566, symmetrized charges were used. This variant of AM1BCC charges is equivalent to OECharges::AM1BCCSymSPt in the Quacpac toolkit, which averages the partial charges of topologically-equivalent atoms.
4. The charged molecule is passed into the Zap toolkit to calculate solvation energy using the ZAP9 parameters for radii and SA.

### OmegaZap: submission #561

The goal of this method is to generate a single conformer with a suitable solution-phase geometry and then use PBSA to calculate the hydration free energy. This submission is based on the best submission presented by Ellingson et al. [11] in SAMPL2 that did not involve expensive QM calculations. This method also tests the hypothesis that a single conformer can be used for calculating transfer energies; however, this method uses a low-energy solution-phase geometry rather than a low-energy gas-phase geometry.

The distinguishing hypothesis of this submission is that OMEGA generates suitable solution-phase geometries due to removing intramolecular electrostatics from the default MMFF94S force field. This can be thought of as an extreme form of a solvent model, similar to a very high solvent dielectric. The lack of electrostatics favors extended conformations that are more accessible for interacting with solvent rather than collapsed conformers that form internal electrostatic interactions. This submission uses the single, lowest-energy conformer from OMEGA and only differs from the SAMPL2 submission in that the new hydrogen sampling option is enabled.

The workflow for this method is as follows:

1. Pass the molecule into OMEGA with hydrogen sampling enabled in order to generate a single conformer.
2. Pass single conformer output from OMEGA into SZYBKI and optimize using default settings.
3. Pass the optimized SZYBKI structure into the *molcharge* application from the QUACPAC package and calculate default AM1BCC charges. The variant of AM1BCC charges used is equivalent to OECharges::AM1BCCNoSym in the Quacpac toolkit, which does an internal AM1 [27] optimization before assigning charges and does not symmetrize bond-topologically equivalent atoms.
4. Pass the charged molecule into the Zap toolkit to calculate solvation energy using the ZAP9 parameters.

### FreeformConf: submissions #567 through #570, #572, and #573

The goal of this method is to generate a complete conformer set for both the gas-phase and solution-phase, create approximate partition functions for these sets, and then calculate the hydration free energy from the partition functions. We discovered afterwards that an unneeded standard-state correction had been applied to these submissions, which significantly altered the results. Remarkably, the unphysical term was beneficial and removing it worsened results by making the transfer energies systematically too negative. Some of these multi-conformer submissions to the SAMPL4 challenge did very well and were not distinguishable from the best methods with statistical certainty; however, the fact that these submissions required an unphysical term in order to do well is puzzling and cause for concern. These results are still under active investigation and there will be no further discussion of them at this time.

### Statistics

In addition to the common statistics of RMS error, mean unsigned error (MUE), and mean signed error (MSE), we performed paired *t*-tests for our submissions. The application of paired *t*-tests was also used in the previous SAMPL challenge [6]. We used IPython [28] notebook with the scipy and numpy packages [29] to calculate paired *t* *-*test *p*-values. All paired *t*-tests were calculated using a standard two-sided null hypothesis, which is that the methods being compared are equivalent.

## Discussion

The submitted values for the hydration free energies are given in Table 1. A complete listing of RMS, mean unsigned, and mean signed errors is given in Table 2. This table is ordered by submission number. Table 3 is ordered by MUE and also gives the paired *t*-test *p*-values for method comparisons. The errors and *p*-values will be referred to in this section.

**Table 1:** The hydration free energies from FreeformSolv, FreeformSolvNoSym, OmegaZap, and the experimental values in kcal/mol. The submission ID is given above the method name

SAMPL ID	565	566	561	Exp
	FreeformSolvNoSym	FreeformSolv	OmegaZAP	
SAMPL4_001	−19.28	−19.40	−24.30	−23.62
SAMPL4_002	−2.70	−2.91	−3.47	−2.49
SAMPL4_003	−4.47	−4.49	−4.98	−4.78
SAMPL4_004	−4.58	−4.59	−5.04	−4.45
SAMPL4_005	−3.44	−3.43	−5.51	−5.33
SAMPL4_006	−4.96	−4.95	−5.13	−5.26
SAMPL4_009	−8.38	−8.89	−9.46	−8.24
SAMPL4_010	−5.21	−5.87	−5.34	−6.24
SAMPL4_011	−8.20	−8.15	−8.42	−7.78
SAMPL4_012	−2.77	−2.84	−2.88	−3.75
SAMPL4_013	−4.55	−4.66	−4.75	−4.44
SAMPL4_014	−3.75	−3.77	−3.88	−4.09
SAMPL4_015	−2.80	−2.89	−3.02	−4.51
SAMPL4_016	−2.97	−3.05	−3.07	−3.20
SAMPL4_017	−2.13	−2.25	−2.28	−2.53
SAMPL4_019	−2.92	−2.93	−3.05	−3.78
SAMPL4_020	−2.72	−2.75	−2.90	−2.78
SAMPL4_021	−9.19	−9.22	−9.17	−7.63
SAMPL4_022	−7.88	−7.91	−7.92	−6.78
SAMPL4_023	−6.24	−6.72	−6.74	−9.34
SAMPL4_024	−5.37	−5.56	−5.91	−7.43
SAMPL4_025	−4.40	−4.47	−4.43	−5.73
SAMPL4_026	−5.61	−5.69	−6.46	−5.31
SAMPL4_027	−2.72	−3.21	−3.56	−4.80
SAMPL4_028	−2.19	−2.61	−3.26	−4.29
SAMPL4_029	−0.11	−0.29	−0.68	−1.66
SAMPL4_030	−2.56	−2.55	−3.23	−2.29
SAMPL4_032	−5.77	−5.77	−5.63	−7.29
SAMPL4_033	−5.71	−6.41	−8.53	−6.96
SAMPL4_034	−5.40	−5.39	−5.58	−5.80
SAMPL4_035	−6.32	−6.32	−9.09	−4.68
SAMPL4_036	−5.42	−5.52	−5.52	−5.66
SAMPL4_037	−5.80	−5.78	−5.97	−5.94
SAMPL4_038	−4.71	−4.71	−4.81	−3.93
SAMPL4_039	−0.65	−0.67	−0.78	−0.85
SAMPL4_041	−3.43	−3.67	−3.65	−5.05
SAMPL4_042	−1.36	−1.50	−1.53	−3.13
SAMPL4_043	0.82	0.82	0.74	0.14
SAMPL4_044	−4.35	−4.21	−4.51	−5.08
SAMPL4_045	−12.34	−12.34	−13.18	−11.53
SAMPL4_046	−9.90	−10.25	−10.70	−9.44
SAMPL4_047	−13.37	−13.32	−13.62	−14.21
SAMPL4_048	−13.20	−13.90	−13.98	−11.85
SAMPL4_049	−4.18	−4.18	−4.48	−3.16
SAMPL4_050	−3.88	−3.88	−4.10	−4.14
SAMPL4_051	−10.58	−10.94	−16.38	−9.53
SAMPL4_052	−3.29	−3.29	−3.57	−2.87

**Table 2:** Root-mean-square error (RMSE), mean unsigned error (MUE), and mean signed error (MSE) in kcal/mol

ID	Name	RMSE	MUE	MSE
561	OmegaZap	1.58 ± 0.32	1.08 ± 0.17	−0.22 ± 0.24
565	FreeformSolvNoSym	1.30 ± 0.18	0.98 ± 0.13	0.48 ± 0.19
566	FreeformSolv	1.23 ± 0.16	0.94 ± 0.12	0.34 ± 0.18

**Table 3:** Paired *t*-test *p*-values for all combinations of our submissions. The table is ordered by mean unsigned error (MUE) in kcal/mol

ID	Name	MUE	Paired *t*-test *p*-value	
			FreeformSolvNoSym	OmegaZap
566	FreeformSolv	0.94	0.33	0.41
565	FreeformSolvNoSym	0.98		0.57
561	OmegaZap	1.08		

### Discussion of single-conformer AM1BCC/PBSA methods

The single-conformer AM1BCC/PBSA methods were among the best performers overall. Only an expensive quantum mechanical method with post-processing of functional groups [30] had a better MUE [1] than FreeformSolv. The MUEs for FreeformSolv, FreeformSolvNoSym, and OmegaZap were 0.94, 0.98 and 1.08 kcal/mol, respectively. All three of these methods were statistically indistinguishable even when accounting for the paired data. There was a surprisingly low paired *t*-test *p*-value of 0.33 for the FreeformSolv and FreeformSolvNoSym calculations. This is lower than the *p*-values that FreeformSolv and FreeformSolvNoSym have with OmegaZap, which are 0.41 and 0.57, respectively, even though there are larger differences in MUE between the methods. All of these *p*-values are too large to reject the null hypothesis of equivalence with statistical certainty.

The MSEs for FreeformSolv and FreeformSolvNoSym were 0.34 and 0.48 kcal/mol, respectively. The MSE for OmegaZap was −0.22, although the uncertainty of ±0.24 does cause the range to overlap zero. As was discussed in the Methods section, the FreeformSolv methods intentionally search for the best gas phase conformer while the OmegaZap intentionally searches for a suitable solution phase conformer. These methods are virtually identical once a single conformer has been chosen; therefore, we conclude that the choice of conformer is the primary cause for the difference in signed errors between these methods.

### Causes of outliers in single-conformer methods

The RMS errors for the single-conformer methods were quite large considering the excellent MUEs. The RMS errors for FreeformSolv, FreeformSolvNoSym, and OmegaZap were 1.23, 1.30 and 1.58 kcal/mol, respectively. These relatively large RMS values were primarily caused by a few catastrophic failures. Due to the similarities between FreeformSolv and FreeformSolvNoSym, for convenience we focus further discussion only on FreeformSolv and OmegaZap.

**Fig. 1:**
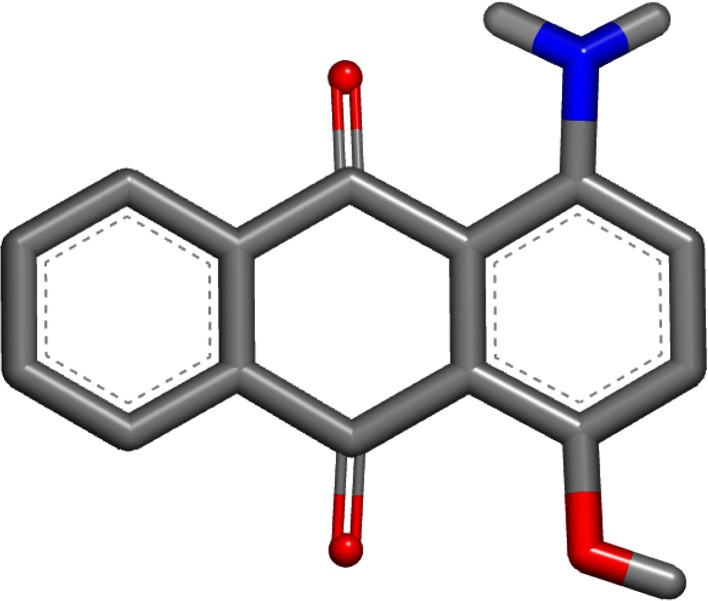
OmegaZap structure for 1-amino-4-hydroxy-9,10-anthraquinone (SAMPL4_051)

**Fig. 2:**
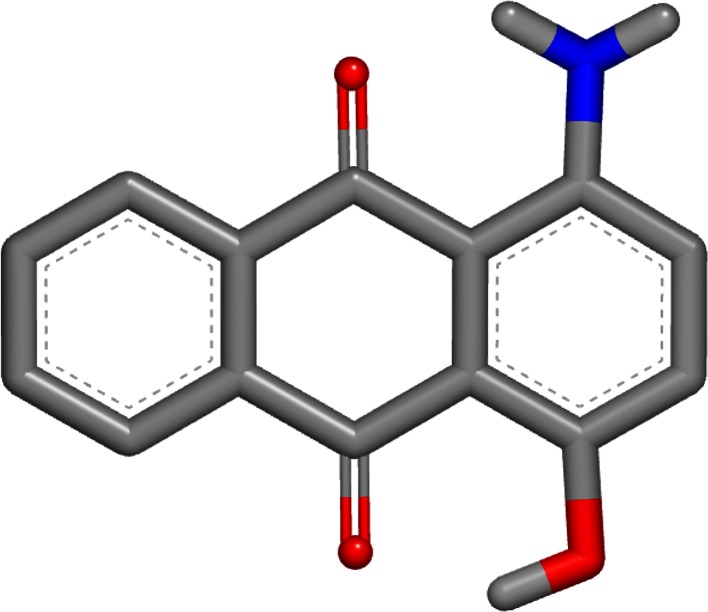
FreeformSolv structure for 1-amino-4-hydroxy-9,10-anthraquinone (SAMPL4_051)

The worst error of these two single-conformer methods was for 1-amino-4-hydroxy-9,10-anthraquinone (SAMPL4_051) with the OmegaZap method. The experimental hydration free energy for this compound was measured to be −9.53 kcal/mol and the OmegaZap result was −16.38 kcal/mol, yielding an error of −6.85 kcal/mol. By contrast, the FreeformSolv result was −10.94 kcal/mol, yielding a much better error of −1.41 kcal/mol. The cause of the large difference in errors is readily apparent when visualizing the OmegaZap and FreeformSolv structures, as shown in Figs. 1 and 2. In the FreeformSolv structure, the hydroxyl rotor is oriented towards the carbonyl in order to make a strong electrostatic interaction, whereas the OmegaZap structure has the hydroxyl rotor oriented away from the carbonyl. The OmegaZap hydroxyl position is an unfortunate consequence of the conformer selection methodology. The disabling of electrostatics causes the very favorable gas-phase conformer to be ignored in favor of the easily solvated conformer. In fact, the conformer selection methodology did exactly what it was designed to do and found a conformer that was over 5 kcal/mol more negative in solvation energy; however, this came at too high a cost in intramolecular electrostatic energy and adversely affected the prediction.

This effect was analyzed using the MMFF94 force field by decomposing the total energy into elastic strain and electrostatic terms. The gas-phase MMFF94 energy differences for FreeformSolv and OmegaZap are given in Table 4 for all of the molecules being discussed in this section. The first data column is the elastic strain energy difference, i.e. stretching and bending terms, torsions, and the van der Waals’ interaction. The second data column is the Coulomb interaction alone. For 1-amino-4-hydroxy-9,10-anthraquinone, the structures have very similar elastic strain energy. However, the Coulomb interaction for this molecule favors the FreeformSolv conformer by an enormous 13.36 kcal/mol in the gas-phase. The OmegaZap conformer was more favorable by 5.44 kcal/mol in solvation energy, but that is clearly not enough to break the strong intramolecular electrostatic interaction.

**Table 4:** The differences in MMFF94 energy between FreeformSolv and OmegaZap in kcal/mol. The first data column is the difference in energy when neglecting the Coulomb term and the second data column is the difference in the Coulomb energy. The third column is the total MMFF94 Energy

Compound name	(FreeformSolv–OmegaZap) MMFF94 Energy		
	Elastic strain	Coulomb	Total
1-amino-4-hydroxy-9,10-anthraquinone	0.61	−13.36	−12.75
Mannitol	2.33	−14.91	−12.58
2-hydroxybenzaldehyde	1.44	−4.83	−3.39

**Fig. 3:**
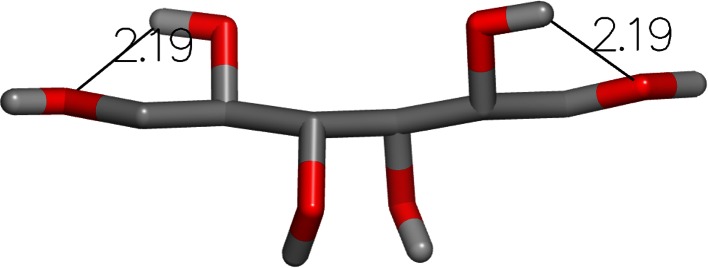
OmegaZap structure for Mannitol (SAMPL4_001) with hydrogen bonds measured in angstroms

**Fig. 4:**
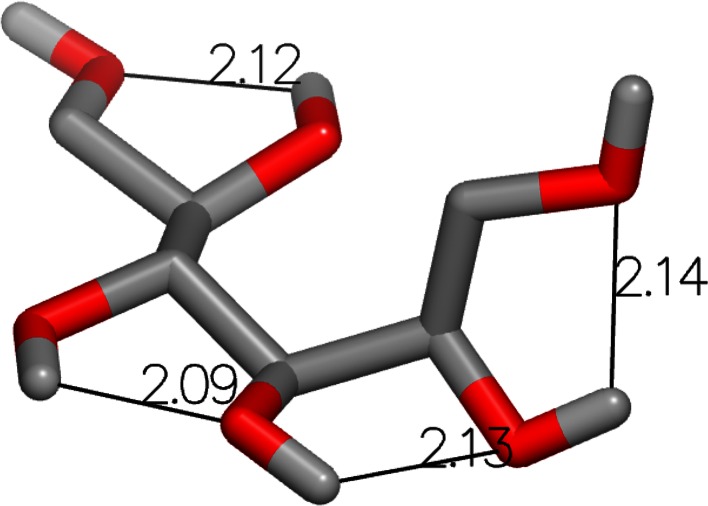
FreeformSolv structure for Mannitol (SAMPL4_001) with hydrogen bonds measured in angstroms

The next largest error was for mannitol (SAMPL4_001) with the FreeformSolv method. The experimental hydration free energy for this compound was measured to be −23.62 kcal/mol and the FreeformSolv result was −19.40 kcal/mol, yielding an error of 4.22 kcal/mol. The OmegaZap result for this compound was −24.30 kcal/mol, yielding a very small error of −0.68 kcal/mol. Once again, the cause of the large difference in errors is readily apparent when visualizing the OmegaZap and FreeformSolv structures, as shown in Figs. 3 and 4. The FreeformSolv structure is much more compact in order to form a chain of hydrogen bonds, whereas the OmegaZap structure is significantly more extended.

As shown in Table 4, the FreeformSolv conformer has more favorable electrostatics by an enormous 14.91 kcal/mol, very similar to the 13.36 kcal/mol in the previous molecule. Yet unlike 1-amino-4-hydroxy-9,10-anthraquinone, the calculated solvation energy for the OmegaZap conformer is very close to the experimental value rather than far too negative. While the FreeformSolv conformer has higher elastic strain energy by 2.33 kcal/mol, it is still 12.58 kcal/mol more favorable when including all MMFF94 terms. Despite the OmegaZap result’s excellent agreement with experiment, we do not have physics-based evidence to claim that the conformer used to model the transfer energy is actually the dominant conformer in solution.

There is no obvious explanation for the poor performance of the FreeformSolv conformer on this molecule. A similar molecule in the SAMPL2 challenge, glucose, was also problematic for this method [7]. One contribution to the large error in glucose was the lack of hydrogen sampling. This resulted in a hydroxyl rotor forming an inferior electrostatic interaction, which in turn yielded a structure that was not the optimal gas-phase conformer. Hydrogen sampling has since been added to the OMEGA algorithm, and enabling this option retrospectively with FreeformSolv has shown that the hydroxyl rotors of mannitol had been oriented optimally. While the OmegaZap conformation is unreasonably high-energy when accounting for all force field terms, it is possible that other well-solvated conformations exist which are low in total energy. Data is sparse for highly flexible molecules such that it is not well known under what circumstances the single-conformer model will break down. Further research is required in order to understand why this method has large errors for sugars.

**Fig. 5:**
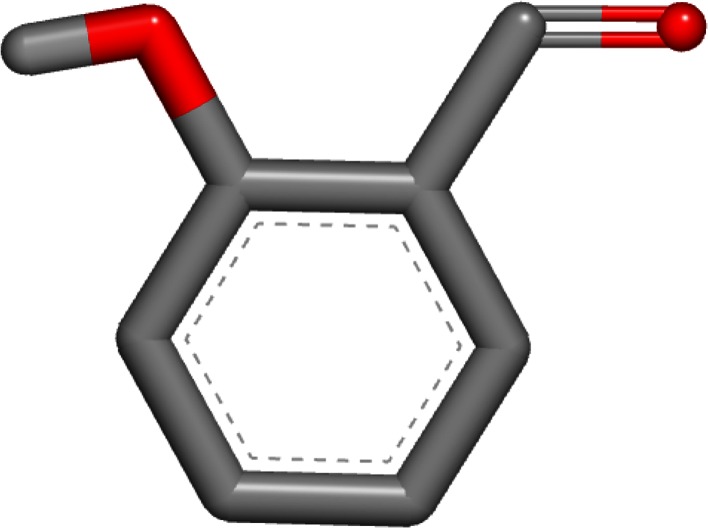
OmegaZap structure for 2-hydroxybenzaldehyde (SAMPL4_035)

**Fig. 6:**
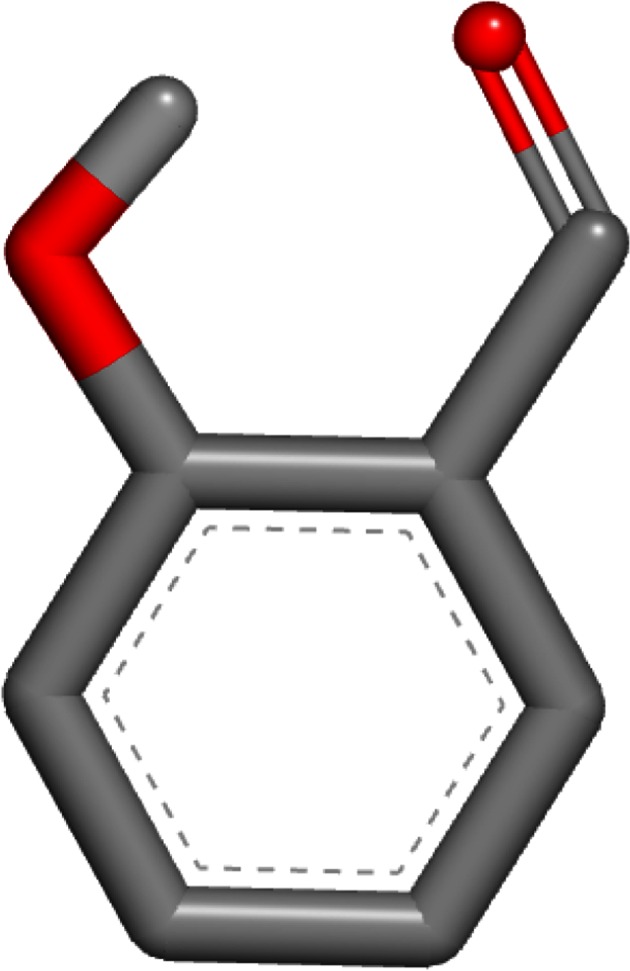
FreeformSolv structure for 2-hydroxybenzaldehyde (SAMPL4_035)

The third and final compound with an unsigned error greater than 3 kcal/mol for either method is 2-hydroxybenzaldehyde (SAMPL4_035). The experimental hydration free energy for this compound was measured to be −4.68 kcal/mol and the OmegaZap result was −9.09 kcal/mol, yielding an error of −4.41 kcal/mol. The FreeformSolv result for this compound was −6.32 kcal/mol, yielding a much better error of −1.64 kcal/mol. The OmegaZap and FreeformSolv structures for this compound are shown in Figs. 5 and 6. In a similar fashion to 1-amino-4-hydroxy-9,10-anthraquinone, the OmegaZap method ignores the very favorable hydroxyl-carbonyl electrostatic interaction and selects a conformer with a more favorable solvation energy.

The MMFF94 energy comparison does not favor FreeformSolv as lopsidedly as the previous two molecules. The FreeformSolv conformation is only more favorable in the gas phase by 3.39 kcal/mol when accounting for all of the force field terms. When considering the favorable solvation effects of the OmegaZap conformer, it is possible that this conformer is actually the dominant species in solution. However, the penalty paid in intramolecular electrostatic energy was not accounted for in the OmegaZap method, thus leading to an error of −4.41 kcal/mol.

### Recognition of likely failures

Detecting and correcting the likely failure cases would have a substantial impact on overall results. If we would have recognized the mannitol failure in FreeformSolv and simply used the OmegaZap result for that compound, the RMS error would have improved from 1.23 to 1.07 kcal/mol. Recognizing the two OmegaZap failures discussed in the previous section and using the FreeformSolv results would have improved the RMS error nearly one-half kcal/mol, from 1.58 to 1.09 kcal/mol.

We have discovered that difference between OmegaZap and FreeformSolv is a good candidate for detecting catastrophic failure cases. Vastly different results for the OmegaZap and FreeformSolv methods indicate that there is a strong conformational dependence on the solvation energy. The conformational dependence is likely caused by strongly interacting polar groups, although it may also be possible that shielding of solvent by nonpolar groups could also cause this effect in large, flexible molecules.

**Fig. 7:**
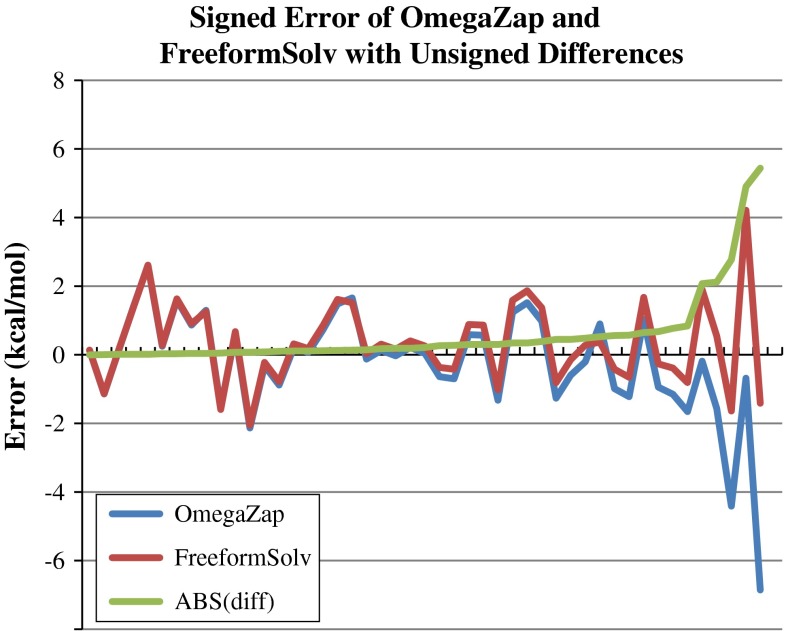
Plot of the signed errors in kcal/mol of the OmegaZap and FreeformSolv submissions for each SAMPL4 compound. The absolute value of the difference between these two methods, labeled ABS(diff), is also shown. The compounds have been ordered by increasing value of ABS(diff) from *left* to *right*

The signed errors of OmegaZap and FreeformSolv along with the absolute value of the differences of the methods is plotted in Fig. 7. The three largest differences between the methods correspond to the three failure cases discussed in the previous section. For the SAMPL4 set, the largest error where OmegaZap and FreeformSolv did not substantially disagree is 2.62 kcal/mol. All three errors larger than this displayed substantial differences between the two methods, indicating that the false negative rate for this metric is low. This effect will need to be studied on a much larger sample set before statistically significant conclusions can be determined.

### Discussion of single-conformer models

In general, our observation from several SAMPL meetings is that our very simple approach does peculiarly well. It is far from clear why a single low-energy conformation should perform as well as it does when there are other available conformations, although the suggestion has been made that polarization causes internally interacting states to be lower in energy that expected [7]. It seems unlikely that the dispersion term in PBSA, i.e. a single number attached to the accessible area of the molecule, is very accurate. In fact, it is known to be misleading when the Van der Waals interactions between solvent and solute are heterogeneous [31]. Some of this variation is parameterized into the radii—for instance, fluorine probably has a large radius in ZAP9 to decrease its contribution to solvation, hence mimicking the lack of dispersion interactions between water and this halide. However, such artificial adjustments are very crude and cannot hope to capture the realities of solvent–solute interaction the way an all-atom simulation might.

One possible explanation is that the experiments are actually less accurate than believed. If this were so, then methods that have more of the physics of solvent interaction than PBSA could be running up against a ‘glass ceiling’, i.e. they cannot get better than simpler methods because of errors in the experimental measurements. In previous SAMPLs, we have seen instances where reexamination of the literature for examples where both all-atom simulations and PBSA had very large errors led to corrections to the proposed experimental value, such as glycerol in SAMPL1 [5]. Several revisions and deletions were made to the current SAMPL4 dataset due to incorrect compounds, reanalysis of the experimental data, and a mistake in a published table reporting experimental data [1, 2]. We have assumed these problems are unusual and that most experimental values have an experimental error as given. However, at this stage it might be worth challenging this assumption. It is very unfortunate for the field that such measurements are no longer routinely made, but it might be possible to examine the literature for instances of difference between experimental groups, or to investigate correlations in errors between theoretical methods to see if these point to systematic biases in any techniques. We have noticed that larger prediction errors seem to correspond to larger solvation energies that are more difficult to measure. In particular, we would welcome new experimental measurements of the solvation energies of sugars that have appeared in recent SAMPL challenges.

## Conclusions

Our submissions using OpenEye’s tools for conformation generation, MMFF94 optimization, AM1BCC charges, and PBSA continuum electrostatics continue to be among the top performers for the SAMPL challenge. The single-conformer AM1BCC/PBSA methods; FreeformSolv, FreeformSolvNoSym, and OmegaZap; all performed well in the SAMPL4 hydration free energy challenge and were among the best performers overall. The paired *t*-test results indicate that none of our AM1BCC/PBSA submissions could be differentiated from each other with statistical certainty. The single-conformer AM1BCC/PBSA methods are also among the least computationally expensive, ranging from 0.5 to 2.0 s/mol on average for the complete calculation.

Both methodologies for single conformer selection are susceptible to catastrophic failures. The best solution-phase conformer can either sacrifice beneficial electrostatic interactions for inferior solvent ones or not properly account for the electrostatic interactions that were given up. The OMEGA algorithm used for the OmegaZap method has been designed to reproduce conformers of bound ligands, which often adopt conformations that allow them to interact with the protein active site in favor of intramolecular interactions. When calculating hydration free energies, the default Omega algorithm may not generate the ideal ensemble from which to pick a low-energy conformer. Further research is required in order to understand the single large FreeformSolv failure. Detecting and correcting these failures would have yielded exceptionally low RMS errors of 1.07 and 1.09 kcal/mol for FreeformSolv and OmegaZap, respectively. A large difference between FreeformSolv and OmegaZap predictions, e.g. greater than 2.5 kcal/mol, is an indication of failure and the corresponding structures should be visually inspected. A more nuanced approach to conformer selection could yield substantial benefits in the future.

## Electronic supplementary material

Below is the link to the electronic supplementary material.

Supplementary material 1 (CSV 3 kb)

Supplementary material 2 (TXT 1 kb)

Supplementary material 3 (PY 1 kb)
